# Recurrence of Decay

**Published:** 1891-06

**Authors:** J. G. Templeton


### "Recurrence of Decay."

J. G. TEMPLETON.

Read before the Northern Ohio Dental Association, Pittsburgh, Pa.

The great Hebrew poet once said in his haste that all men were liars, but if he were now living he might take his time, and say deliberately that most, if not all dentists fail to prevent recurrence of decay in the teeth of all for whom they operate. The latter is a plain statement of facts, however much we may dislike to see our best operations undermined by the dissolving of the lime salts of a tooth, or acknowledge that we have not exercised sufficient care to make contour fillings that will stand the test as "Before Decay's effacing fingers Had swept the lines where Beauty lingers." The occurrence of recurrent caries may be brought about by neglect to observe hygienic care of the teeth after operations for their preservation. Such a class of clients, of course, always expect their dentist to control the laws of nature. We will here mention a few of the causes of recurrent decay, viz: zymotic diseases, impaired nutrition, gestation and lactation and careless and imperfect manipulation by dentists.

A zymotic or exanthematous disease is one in which "there is some morbific principle acting on the organism similar to a ferment," and includes all eruptive fevers and cutaneous diseases, the teeth being affected on account of their connection with, or being a part of the tegumentary or dermal system.

That the teeth are likely to decay when the nutritive functions of the system are impaired, goes without saying.

In gestation and lactation the mother's system is so largely drawn upon by the increased demand for the phosphate of lime that the teeth fail to receive their accustomed supply. Hence, in the teeth of such of our patrons we find new solutions of continuity and recurrence in proximity to fillings.

Recurrence is very often due to lack of care or thoroughness where most needed. Such places are the cervical walls, particularly in bicuspids and molars, also the side of the cavity next to the operator. Special attention must be given these points, both in the preparation of the cavity and in packing the gold, otherwise there will be imperfect adaptation of the filling with the walls, leaving small spaces. However minute these spaces may be they will be sufficient to permit the ingress of moisture and recurrence will ensue.

Great care is likewise to be observed in packing gold against the walls of any part of the cavity to prevent the point of the plugger from coming in contact with the tooth structure.

The cervical wall should be covered with heavy tin foil and condensed or packed down with smooth-pointed instruments, thus securing the best adaptation, and also the preservative qualities of tin at the point most likely to be attacked.

Approximal cavities of bicuspids and molars should be prepared so that there will be no contact of tooth surfaces after the fillings are finished.

Our duty is to do all we can to prevent, and yet we can not control the laws of nature because *change* is written on her face. Hence, oft times we'll see where least desired or expected the " Recurrence of Decay."

### Recurrence of Decay.

